# Supplementary material for: A constricted opening in Kir channels does not impede potassium conduction
Source: Nat Commun. 2020 Jun 15;11:3024. doi: 10.1038/s41467-020-16842-0 (PMC7295778; doi:10.1038/s41467-020-16842-0)
Supplement: Supplementary file 1 — Supplementary Information [file 41467_2020_16842_MOESM1_ESM.pdf]

The constricted pore of Kir channels does not impede conduction of potassium ions
Black et al

SUPPLEMENTARY INFORMATION

Figures 1 to 9

Tables 1 to 5

Supplementary Figure 1.

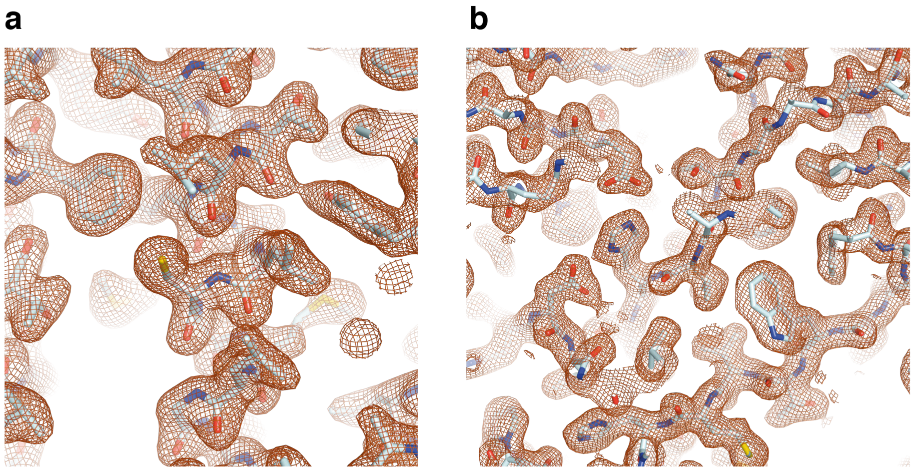

Supplementary Figure 1. The quality of the 2.0 Å KirBac3.1 crystal structure for use as a starting model for MD simulations is indicated by representative $2|F_o|-|F_c|$ electron density calculated using coefficients from the final refined model. Fine mesh is contoured at 1.0 σ. Representative electron density in (**a**) transmembrane pore and (**b**) cytoplasmic assembly. The protein is included as a stick representation.

Supplementary Figure 2.

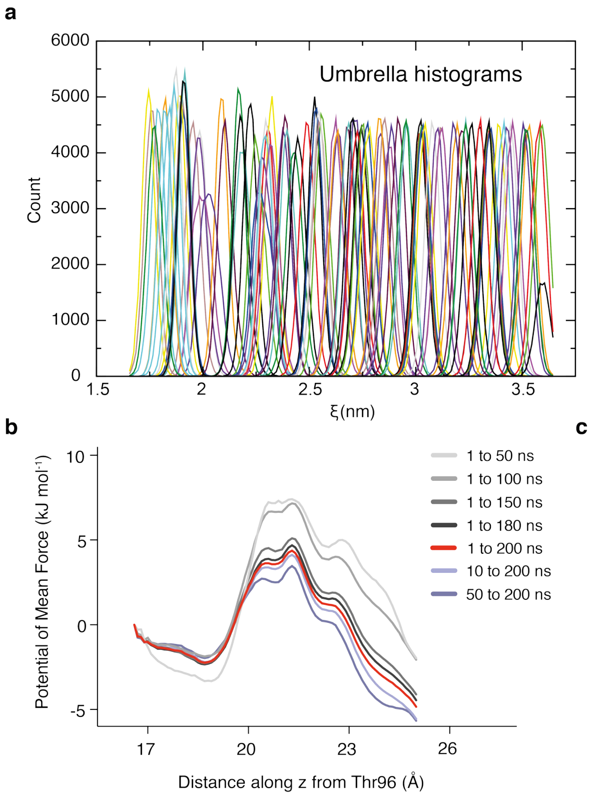

c

Simulation Period (ns)	PMF (kJ mol^{-1})
1 to 50	10.7
1 to 100	9.0
1 to 150	7.3
1 to 180	7.0
1 to 200	6.6
10 to 200	6.3
50 to 200	5.4

Supplementary Figure 2. Optimisation of parameters for PMF calculations. The umbrella sampling method was utilised in calculating the potential of mean force (PMF) experienced by a K^+ ion as it moves along the molecular axis. Each umbrella sampling simulation contains 95 individual sampling windows, with each window simulated for 200 ns. To explore the PMF calculation outcomes, periods of umbrella sampling simulation were first optimised. (**a**) 95 histograms were extracted from umbrella simulations. (**b**) The PMFs are shown as a function of the distance along the z-direction between a K^+ cavity ion 'pulled' along the z-axis relative to the center of mass of the four Thr96 residues. Lines of different colours represent the PMF calculated over different sampling periods. The red line represents the PMF from a simulation in the period 1 to 200 ns in each window, as in Fig. 2A of the manuscript. Other lines represent the PMF of simulation periods of increasing length (1 to 50 ns, 1 to 100 ns, 1 to 150 ns, 1 to 180 ns), or with a small equilibration period truncated (10 to 200 ns and 50 to 200 ns). The position of the largest free energy barrier is comparable in each. (**c**) The free energy barriers extracted from PMF values obtained by different simulation periods. The free energy barrier decreases as the simulation time increases from 50 to 200 ns for each window, as greater sampling of the conformational space allows the calculation to converge. Allowing for initial equilibration time by removal of the first period of 1, 10 or 50 ns also decreases the peak value within a magnitude range of 1 kJ mol^{-1}, which lies within the region of uncertainty (±2 kJ mol^{-1}) of the WHAM calculations. The data presented here indicates that with the choice of parameters utilized, simulations were well equilibrated and that the conformational space sampled by umbrella sampling was both self-consistent and sufficient to allow convergence.

Supplementary Figure3.

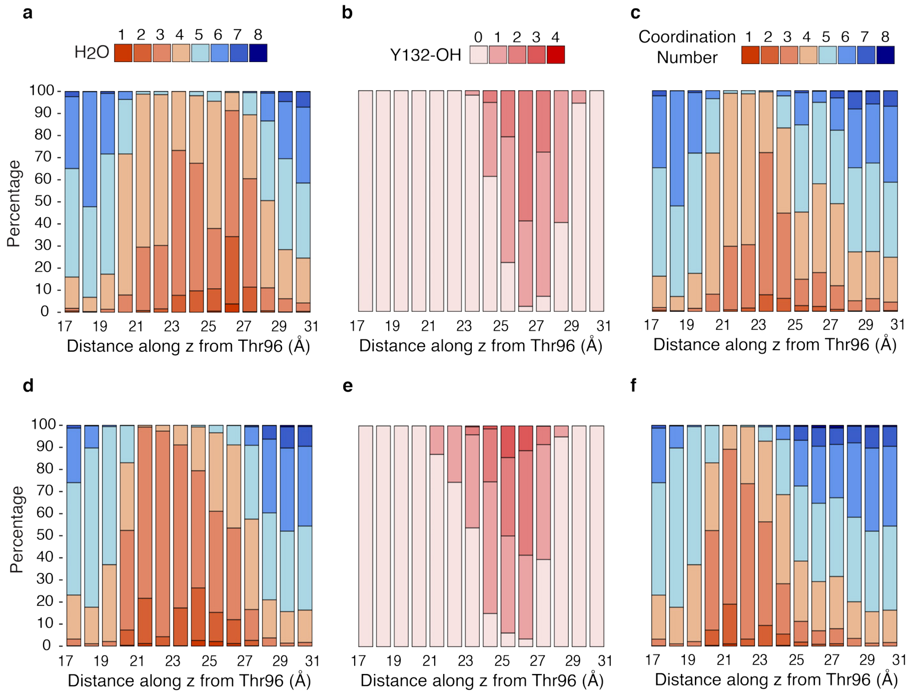

Supplementary Figure 3. Normalized histograms enumerate the number of oxygens coordinating K^+ as a function of distance from Thr96. Data are accumulated from umbrella sampling simulations of wild type KirBac3.1. Each bin represents the number of ligands within 3.0 Å of K^+, expressed as a percentage. Panels A to C represent wild type KirBac3.1 and panels D to E the S129C-F135C disulfide-linked channel. (**a/d**) Water molecules are represented (**b/e**) The hydroxyls of Tyr132 are represented. (**c/f**) The sum total of water and hydroxyl ligands, as shown in Figure 2.

Supplementary Figure 4. Statistical analysis of small fluctuations in the internuclear distances (D; D1 and D2) between diagonally opposed Tyr132 hydroxyl oxygens. Plotting D1-D2 couplets from 100,000 simulation structures against the two contributing cross-sectional diagonals reveals a profile with peaks at strongly favoured configurations (equating to low free energy states). (**a**) An asymmetric simulation intermediate of KirBac3.1 shown in a similar perspective to Fig. 1B introduces the two diagonals, D1 and D2, that define a D1-D2 couplet. (**b**) Hexagonal bin plots of D1 against D2 for structures extracted from unrestrained MD (20 simulations, 1 μsec in total) performed at a field strength of 50 mV nm^{-1}. Structures were distributed into hexagonal bins on the basis of their D1-D2 couplets. Distinct peaks in the continuum occur centrally (CP), where D1 and D2 are equivalent (4-fold symmetry), and peripherally (PP), where one diagonal has lengthened at the expense of the other, approximating 2-fold symmetry. The count in each bin is described by a colour key, with dark purple representing the greatest number of structures and beige the least. The predominant central peak reflects a pore diameter of about 6 Å, directly comparable to the tyrosine collar in the 2.0 Å crystal structure; a less prominent CP is also present at 8 Å (**c**) As for panel B, but with simulations carried out at the higher transmembrane potential of 100 mV nm^{-1}. The increase in field strength led to a sparser distribution of peaks. The peripheral peaks and the 8 Å CP gain in intensity while the 6 Å CP peak diminishes. (**d**) A representative simulation structure of the KirBac3.1 pore (residues 33:138) from CP of panel C. It is shown as a solvent accessible surface of the cytoplasmic face of the pore; the hydroxyl oxygens of Tyr132 feature in red. Note the approximate 4-fold molecular symmetry of the aperture. (**e**) A representative PP structure showing that the Tyr132 collar adopts approximate 2-fold symmetry with opening ablated. (**f**) Using data from 11 short simulations, each corresponding to ~2.5 ns either side of the Tyr-OH 'plane', the couplets of structures at the precise moment of passing the plane are plotted as red dots. The interchangeable D2-D1 couplets are shown as yellow dots.

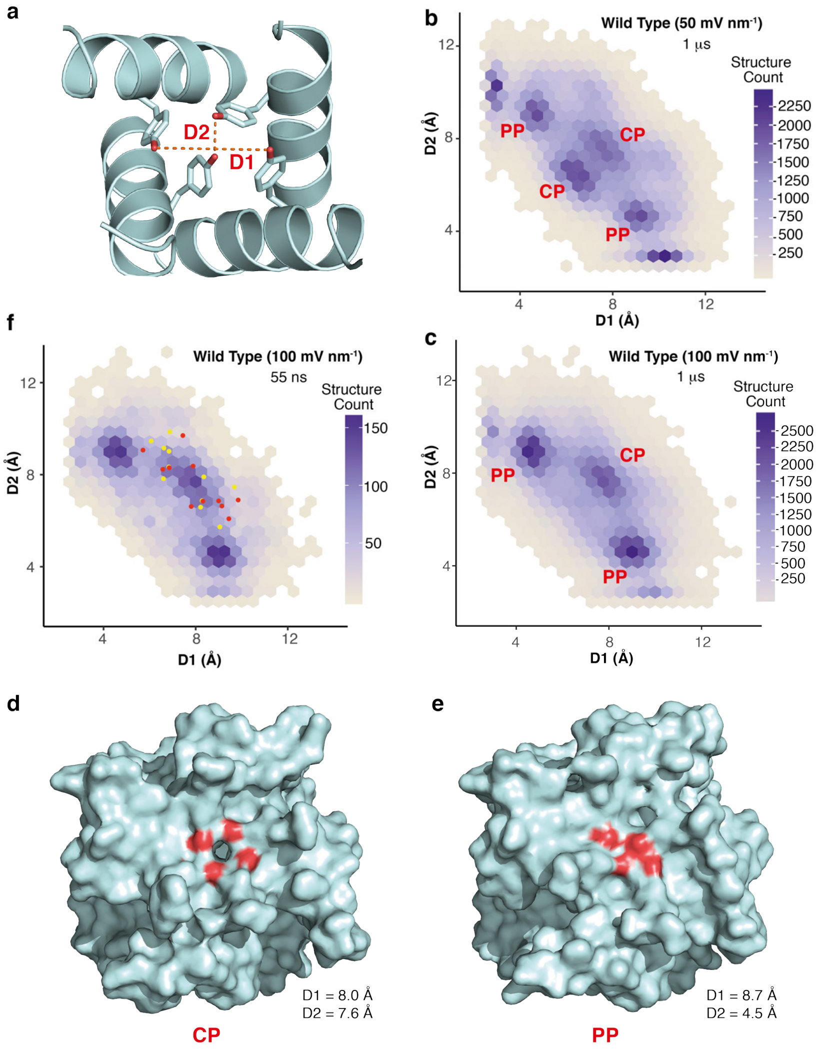

Supplementary Figure 5.

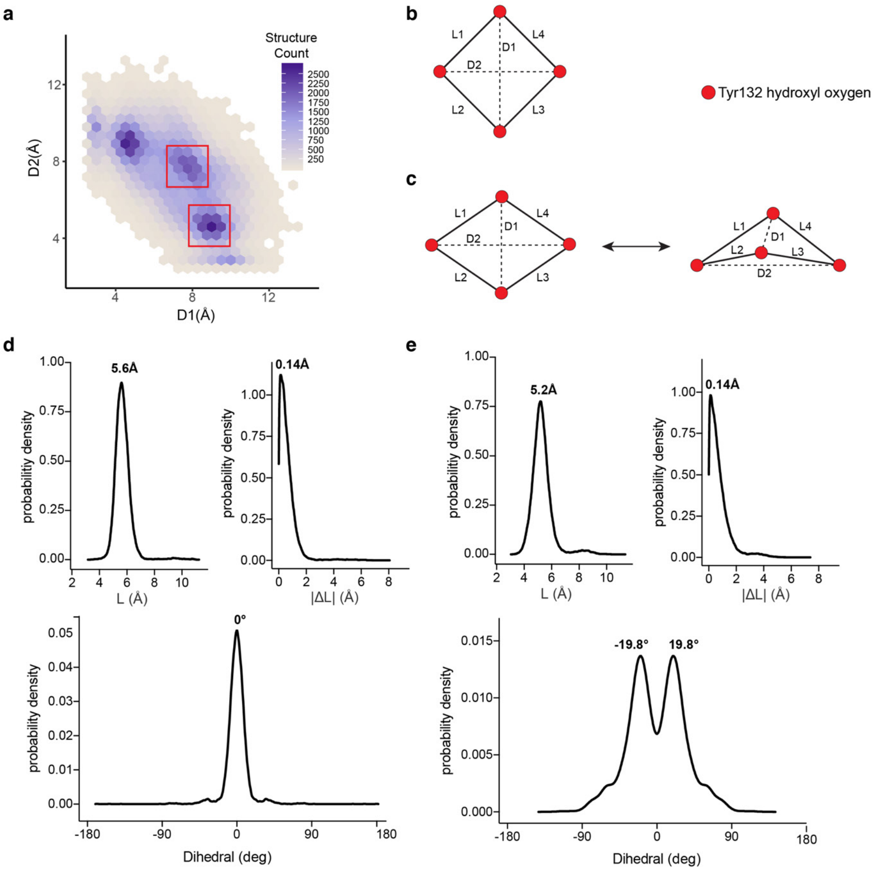

Supplementary Figure 5. Analysis of the central (CP) and peripheral (lower PP) peaks in the hexbin D1-D2 couplet distribution indicates interdependent motion of the four tyrosine hydroxyls. (**a**) Hexagonal bin plot as in Supplementary Figure 4. (**b**) Schematic geometry of the Tyr132-OH oxygens of CP. Each structure has two diagonals, D1 and D2, and four sides of length L1 to L4. A square configuration depends on equivalence in D1 and D2, and in L1 – L4, with all oxygen atoms in plane. (**c**) In PP, the diagonals D1 and D2 are not equivalent and the atoms form tetrahedrons where D1 and D2 rarely intersect; a tetrahedral configuration is depicted on the right and a rhomboid intermediate on the left. (**d**) On the left is plotted the probability density analysis for L (L1, L2, L3 and L4) of structures within the boxed CP area; the mode of the integrated distribution is a sharp peak at 5.6 Å. On the right is the corresponding probability density distribution for ΔL CP (where ΔL includes all values of L1-L2, L1-L3, L1-L4, L2-L3, L2-L4 and L3-L4). The ΔL mode at 0.14 Å is sharp, signifying all L values are of approximately equal length, while a dihedral of 0° indicates that the four oxygen atoms are coplanar. The data verify that CP hydroxyls are arranged in a square (as in panel b) which, in the context of the channel tetramer, implies C4 symmetry. (**e**) The probability densities for the boxed PP area, where D1 and D2 are unequal, show that the L values are equivalent, with mode values of 5.2 Å for L and 0.14 Å for ΔL. The dihedral distribution is both broader and bimodal, with peaks at +/- 20°, reflecting the schematic in panel E. By distinguishing PP geometry from the alternative (*i.e.* that L1-L4 are inequivalent), the data infer interdependent motion of the four tyrosine hydroxyls.

Supplementary Figure 6.

a

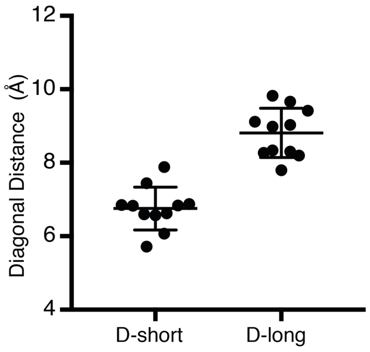

b

	D1	D2	V1	V2
1	5.7	9.0	2.9	6.2
2	6.9	8.3	4.1	5.5
3	7.9	8.3	5.1	5.5
4	7.8	6.6	5.0	3.8
5	9.8	6.9	7.0	4.1
6	9.4	6.1	6.6	3.3
7	6.6	8.2	3.8	5.4
8	7.4	9.6	4.6	6.8
9	9.0	6.8	6.2	4.0
10	9.1	6.6	6.3	3.8
11	8.3	6.8	5.5	4.0

c

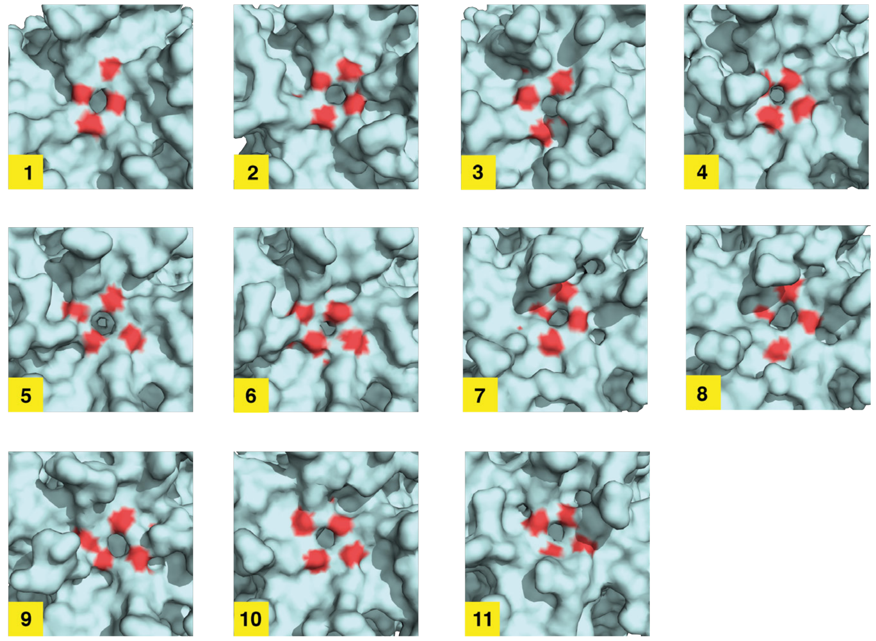

Supplementary Figure 6. The four Tyr132 hydroxyls are asymmetrically disposed at the instant that K^+ ions pass them. (**a**) Statistical analysis of aperture shape as individual K^+ ions pass the tyrosine collar. Each of 11 data points arises from a 5 ns simulation, corresponding to approximately 2.5 ns either side of the Tyr-OH 'plane'. The shorter and longer diagonals of the D1-D2 couplets are extracted and plotted separately, with average values of 6.8 and 8.8 Å, respectively. Data are shown as mean +/- SD. (**b**) The diagonal internuclear distances (D1 and D2) of each of the 11 couplets are given, along with the equivalent van der Waals spacings between the hydroxyl oxygens of Tyr132 (V1 and V2). (**c**) The solvent accessible surface of the intracellular face of KirBac3.1 is depicted in close-up for the 11 simulation structures. The hydroxyl oxygens of Tyr132 indicated in red denote the size and shape of the intracellular aperture at the moment K^+ passes through. All show a small, mildly asymmetric, opening; none are as isotropic as CP or as distorted as PP (see Figs. S4, S5).

Supplementary Figure 7.

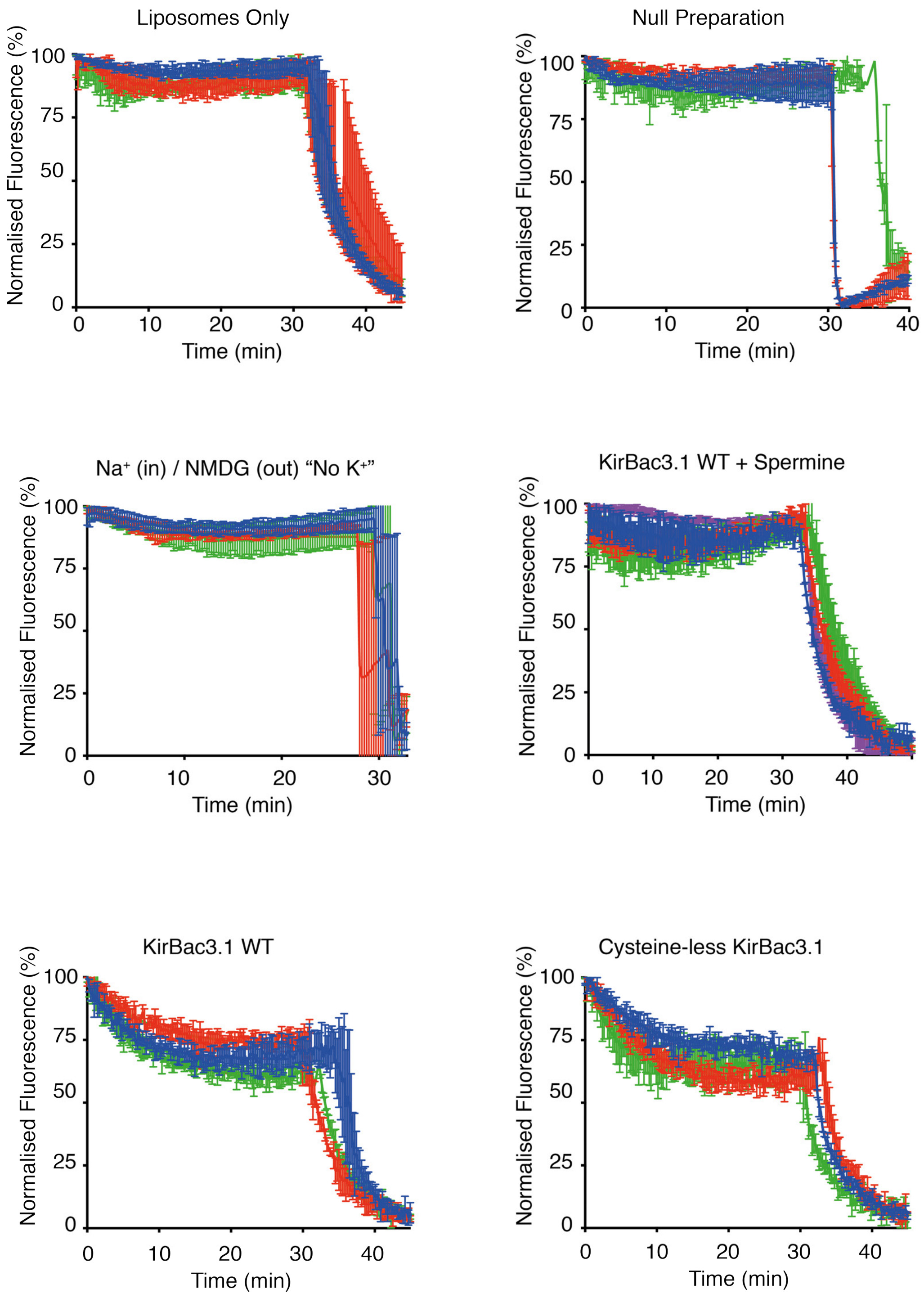

Supplementary Figure 7. Normalised data from liposomal fluorescence flux assays. All normalised data measured for the liposomal flux assay controls are presented here, with the exception of the channel-free liposomes, for which only three independent replicates are shown for clarity. The title above each graph indicates the experimental condition tested. For each experiment, each colour (red, blue, green) represents an independent reconstitution. The standard deviations (of three technical replicates) are indicated by error bars.

Supplementary Figure 8.

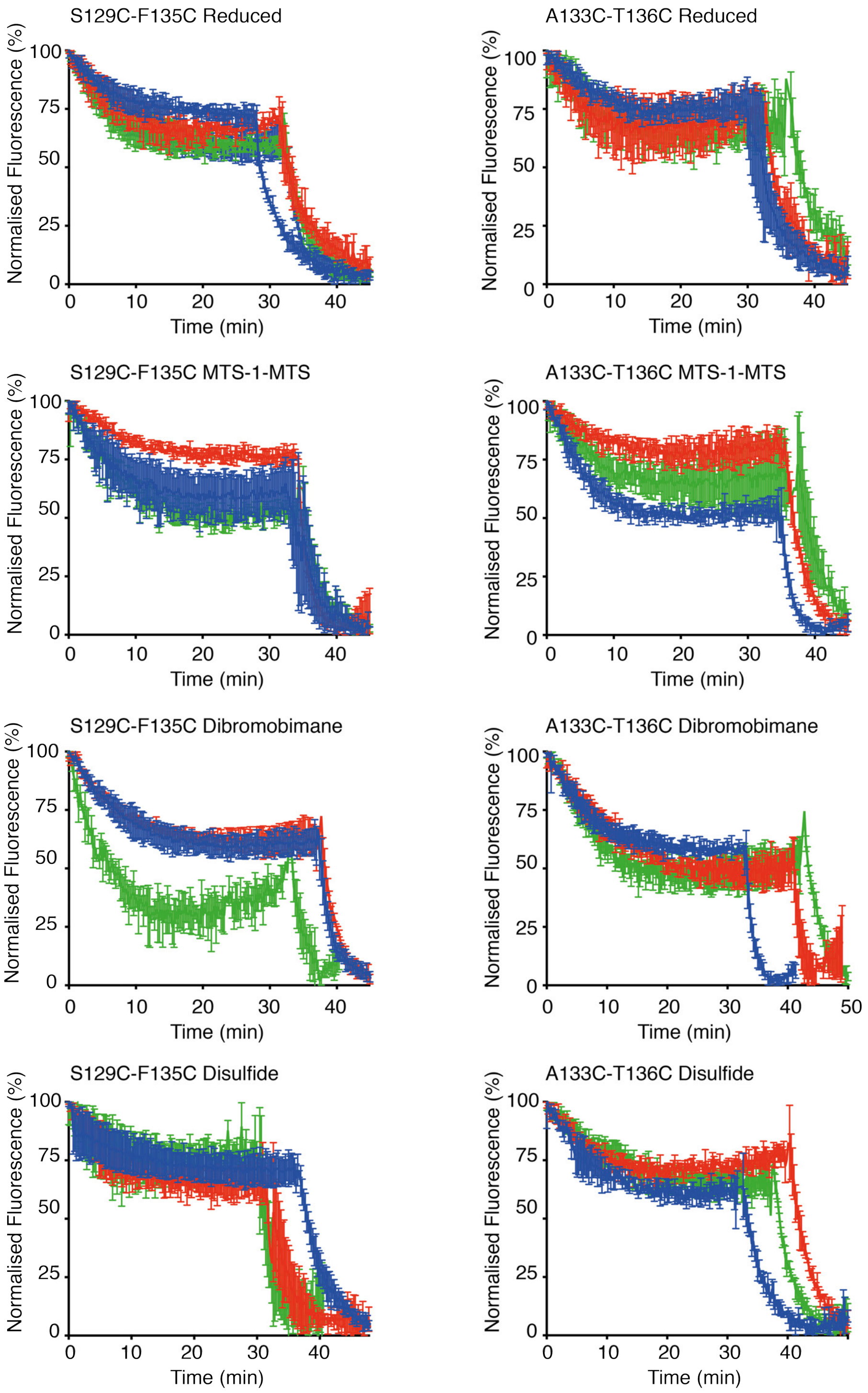

Supplementary Figure 8. Normalised data from liposomal fluorescence flux assays of the Cys-pair mutants. All normalised data measured for these assays are presented here. The title above each graph indicates the experimental condition tested. For each experiment, each colour (red, blue, green) represents an independent reconstitution. The standard deviations (of three technical replicates) are indicated by error bars.

Supplementary Figure 9.

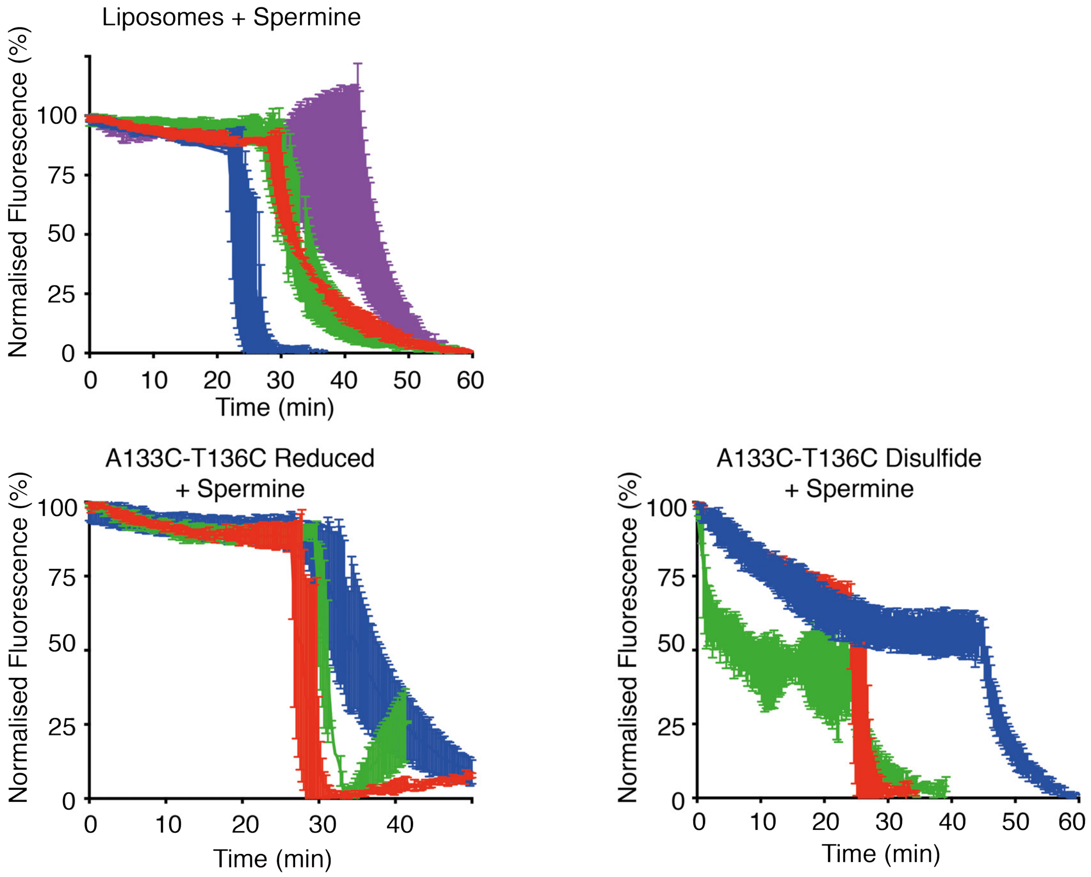

Supplementary Figure 9. Normalised data from liposomal fluorescence flux assays of the spermine experiments. All normalised data measured for these assays are presented here. The title above each graph indicates the experimental condition tested. For each experiment, each colour (red, blue, green, magenta) represents an independent reconstitution. The standard deviations (of three technical replicates) are indicated by error bars.

Supplementary Table 1. Crystallographic Structure Determination and Refinement.

**Data collection**	Wild Type	S129C-F135C MTS-1-MTS	A133C-T136C DBB
Processing system	Denzo/Scalepack	XDS	XDS
Space group	$P42_12$	$P2_12_12$	$P42_12$
Monomers per asymmetric unit	1	2	1
Cell dimensions (Å)	*a* 105.955, *c* 89.639	*a* 83.299, *b* 114.732, *c* 89.464	*a* 103.077, *c* 89.247
Wavelength (Å)	1.0000	0.9537	0.9537
Resolution range (Å)	45.61/2.00	48.3-3.1	46.10-4.0
Unique reflections	35,077	16,064	4,381
R_{merge} (overall/outer shell) [a]	0.111/---	0.218/2.157	0.146/1.812
I/σI (overall/outer shell)	14.97/1.24	12.6/1.7	8.24/1.03
Completeness (overall/outer shell)	0.998/1.00	0.990/0.986	0.990/0.993
Redundancy (overall/outer shell)	6.3/6.3	11.5/11.7	6.99/7.1
$CC_{1/2}$ (overall/outer shell)[b]	N. A.	0.999/0.611	99.9/32.9

**Data refinement**			
Program	Phenix	Phenix	Buster
Resolution range (Å)	45.61/2.00	44.73-3.10	46.11-4.01
Reflections (refine/test)	35,077 (F^+, F^-) 65,700/3311	16,034/835	4,145/195
R factor (R_{cryst}/R_{free})[c]	0.202/0.227	0.248/0.294	0.246/0.273[c]
Mean isotropic *B*-factor B_{iso} ($Å^2$)	39.8	92.4	219.3
RMSD Bond lengths (Å)	0.003	0.003	0.01
RMSD Bond angles (°)	0.546	0.706	1.19
PDB code	**6O9U**	**6O9V**	**6O9T**

[a] $R_{merge} = \sum| I_{obs} - I_{calc}| / \sum I_{obs}$

[b] $CC_{1/2}$ = percentage of correlation between intensities from random half-datasets. Outer shell values are significant at the *p*=0.1% level[1].

[c] These values are R_{xpct}: expectation value of the crystallographic R-factor[2] as reported by autoBUSTER[3].

1. Karplus, P. A. & Diederichs, K. Linking crystallographic model and data quality. *Science* **336,** 1030–1033 (2012).
2. Blanc, E. *et al.* Refinement of severely incomplete structures with maximum likelihood in BUSTER–TNT. *Acta Crystallogr D Biol Crystallogr* **60,** 2210–2221 (2004).
3. Bricogne, G., Blanc, E., Brandl, M., Flensburg, C. & Keller, P. *autoBUSTER, Version 1.6. 0*. (Global Phasing Ltd, 2011).

Supplementary Table 2. Summary of Molecular Dynamics Simulations.

**Equilibration**			
KirBac3.1 condition	Lower cavity		Simulation length (ns)
Wild Type	K^+		101.5
Wild Type	Spermine		101.5
S129C-F135C	K^+		101.5
A133C-T136C	Spermine		101.5
**Steered Molecular Dynamics**			
KirBac3.1 condition	Lower cavity	Pulling Speed (Å/ns)	Simulation length (ns)
Wild Type	K^+	0.15	120
Wild Type	Spermine	0.15	150
S129C-F135C	K^+	0.15	120
A133C-T136C	Spermine	0.15	150
**Umbrella Sampling**			
KirBac3.1 condition	Lower cavity	Number of windows × Simulation length per window (ns)	Simulation length (ns)
Wild Type	K^+	97 × 200	19400
Wild Type	Spermine	106 × 200	21200
S129C-F135C	K^+	115 × 200	23000
A133C-T136C	Spermine	126 × 200	25200
**Unrestrained MD**			
KirBac3.1 condition	Electrical field (mV nm^{-1})	Number of simulations × Simulation length (ns)	Simulation length (ns)
Wild Type + K^+	50	20 × 50	1000
Wild Type + K^+	100	20 × 50	1000
Wild Type + Spermine	0	20 × 50	1000
Wild Type+ Spermine	25	20 × 50	1000
Wild Type+ Spermine	50	20 × 50	1000
**Total simulation time (ns)**			**94746**

Supplementary Table 3. Summary of mass spectrometry data analysing cross-linking of KirBac3.1. The final column shows the expected mass difference (Daltons) if the tetramer has four crosslinks. SD = standard deviation.

Protein	Calculated mass of monomer (after removal of the N-terminal Met)	Observed mass of monomer ± SD	Calculated mass of tetramer	Observed mass of tetramer ± SD	Δ Mass ± SD	Δ Mass
WT	33606.6	33604.74 ± 2.12	134426.4	134419.35 ± 0.35	-7.05 ± 0.35	0
Cysteine-less	33582.5	33580.93 ± 2.24	134330	134324.20 ± 0.66	-5.80 ± 0.66	0
129C-135C reduced	33554.5	33553.80 ± 1.50	134218	134211.84 ± 6.60	-6.16 ± 6.60	0
129C-135C DBB	33554.5	Not Observed	134218	134962.50 ± 8.66	744.50 ± 8.66	756
129C-135C S-S	33554.5	Not Observed	134218	134209.33 ± 10.30	-8.67 ± 10.30	-8
129C-135C M1M	33554.5	Not Observed	134218	134510.95 ± 10.63	292.95 ± 10.63	304
133C-136C reduced	33616.6	33612.49 ± 3.54	134466.4	134457.20 ± 2.55	-9.20 ± 2.55	0
133C-136C DBB	33616.6	Not Observed	134466.4	135219.50 + 0.15	753.10 ± 0.15	756
133C-136C S-S	33616.6	Not Observed	134466.4	134465.21 ± 2.57	-1.19 ± 2.57	-8
133C-136C M1M	33616.6	Not Observed	134466.4	134794.24 ± 6.17	327.84 ± 6.17	304

Supplementary Table 4. Summary statistical data for functional assays on control, wildtype and mutant KirBac3.1

Condition	Mean fluorescence change prior to valinomycin addition ± SEM	Independent replicates (technical replicates for each experiment)	Significance compared to Channel-Free[a]		Significance compared to Wild Type[a]	
Liposomes Only (Channel-Free)	8.15 ± 0.74	8 (3, 4, 6, 5, 3, 3, 5, 3)			***	0.003
Wild type KirBac3.1	32.7 ± 3.6	3 (3, 3, 3)	***	0.003		
Cysteineless KirBac3.1	37.3 ± 4.3	3 (4, 3, 3)	****	< 0.0001	ns	0.9908
Wild type spermine block (500 μM / 1000 μM)	8.71 ± 1.0	4 (2, 3, 3, 4)	ns	>0.9999	**	0.0020
No K^+ Control (Na^+_i/$NMDG^+_o$)	10.2 ± 1.8	3 (3, 3, 3)	ns	0.9995	**	0.0085
KirBac3.1 Null plasmid	8.9 ± 0.5	3 (3, 3, 3)	ns	0.9998	**	0.0014
129C-135C Reduced	36.3 ± 3.5	4 (3, 3, 3, 3)	****	<0.0001	ns	0.9990
129C-135C Bimane	51.8 ± 9.7	3 (3, 3, 6)	****	<0.0001	*	0.0377
129C-135C Disulfide	27.5 ± 5.4	3 (3, 3, 6)	**	0.0072	ns	0.9899
129C-135C MTS-1-MTS	39.6 ± 7.7	3 (3, 6, 3)	****	<0.0001	ns	0.9957
133C-136C Reduced	32.1 ± 2.8	3 (4, 3, 3)	***	0.0005	ns	0.9999
133C-136C Bimane	54.0 ± 5.1	3 (3, 3, 3)	****	<0.0001	*	0.0149
133C-136C Disulfide	40.2 ± 3.7	3 (3, 3, 3)	****	<0.0001	ns	0.8414
133C-136C MTS-1-MTS	37.1 ± 9.0	3 (3, 3, 3)	***	0.0001	ns	0.9957
133C-136C Disulfide spermine block (500 μM / 1000 μM)	49.3 ± 3.8	3 (3, 3, 4)	****	<0.0001	ns	0.0943
133C-136C reduced spermine block (500 μM / 1000 μM)	13.2 ± 1.3	3 (6, 4, 3)	ns	0.9900	*	0.0311
Channel-free spermine block (500 μM / 1000 μM)	8.1 ± 1.6	4 (3, 3, 4, 3)	ns	>0.9999	**	0.0014

[a]Significance, as calculated from Dunnett's multiple comparisons test, is indicated for each dataset compared to channel-free liposomes (Column 5) and wild type proteoliposomes (Column 6). (ns = not significant, * = p ≤ 0.05, ** = p ≤ 0.01, *** = p ≤ 0.001, **** ≤ 0.0001)

Supplementary Table 5. Summary rates data for functional assays

Condition	Mean rate (time constant, tau) ± SEM (min)	Mean rate (rate constant, K) ± SEM (s^{-1})	Independent replicates (technical replicates for each experiment)
Wild Type KirBac3.1	6.56 ± 0.57	0.0026 ± 0.0002	3 (3, 3, 3)
Cysteineless KirBac3.1	6.40 ± 1.46	0.0030 ± 0.0009	3 (4, 3, 3)
129C-135C Reduced	6.21 ± 0.20	0.0027 ± 0.0001	4 (3, 3, 3, 3)
129C-135C Bimane	6.29 ± 1.02	0.0028 ± 0.0005	3 (3, 3, 6)
129C-135C Disulfide	5.46 ± 1.26	0.0035 ± 0.0009	3 (3, 3, 6)
129C-135C MTS-1-MTS	7.26 ± 0.63	0.0023 ± 0.0002	3 (3, 6, 3)
133C-136C Reduced	5.57 ± 0.97	0.0032 ± 0.0006	3 (4, 3, 3)
133C-136C Bimane	8.98 ± 1.95	0.0020 ± 0.0003	3 (3, 3, 3)
133C-136C Disulfide	8.65 ± 2.05	0.0021 ± 0.0004	3 (3, 3, 3)
133C-136C MTS-1-MTS	5.97 ± 0.38	0.0028 ± 0.0002	3 (3, 3, 3)
ALL DATASETS	6.73 ± 1.04	0.0027 ± 0.0005	31
