# Supplementary material for: A constricted opening in Kir channels does not impede potassium conduction
Source: Nat Commun. 2020 Jun 15;11:3024. doi: 10.1038/s41467-020-16842-0 (PMC7295778; doi:10.1038/s41467-020-16842-0)
Supplement: Supplementary file 3 — Description of Additional Supplementary Information Files [file 41467_2020_16842_MOESM3_ESM.pdf]

Description of Additional Supplementary Files

File Name: Supplementary Movie 1

Description: Molecular dynamics simulations reveal the partial dehydration and rehydration of a K+ ion as it permeates the tyrosine collar. The KirBac3.1 pore is depicted as a ribbon diagram with transparent solvent accessible surface. As the movie starts, the front and back subunits are peeled away for viewing clarity. The Tyr132 side chain is present in stick representation, and a nearby K+ ion as a green vdW sphere. Water molecules within the pore cavity at the commencement of the simulation are represented in stick (liquorice) form, while water molecules initially present in the cell are shown as vdW spheres. During the simulation, only water molecules within 3.6 Å of the K+ ion become visible. The movie shows progress of a single K+ ion from the lower part of the cavity into the cell. The K+ hydration state drops from seven to four inner cavity waters as it passes Cb of Tyr132. As it passes the tyrosine hydroxyls, the partial hydration shell is consecutively and progressively replaced with intracellular water molecules until the ion is hydrated with seven water molecules arising from the cell.
